# Telemedicine Acceptability and Experience in a Marginalized Population at Risk for Hepatitis C Virus

**DOI:** 10.1016/j.gastha.2023.09.010

**Published:** 2023-09-28

**Authors:** Rebecca G. Kim, Claire McDonell, Sarah Burbank, Meghan D. Morris, Jennifer C. Price

**Affiliations:** 1 Division of Gastroenterology and Hepatology, Department of Internal Medicine, University of Utah, Salt Lake City, Utah; 2 Department of Epidemiology and Biostatistics, University of California San Francisco, San Francisco, California; 3 Department of Medicine, University of California San Francisco, San Francisco, California; 4 Division of Gastroenterology and Hepatology, Department of Medicine, University of California San Francisco, San Francisco, California; 5 Liver Center, University of California San Francisco, San Francisco, California

Hepatitis C virus (HCV) incidence is rising in the United States, especially among young persons who inject drugs.^1^^,^^2^ Innovative approaches to diagnose and treat HCV have been proposed^3^; however, these efforts were derailed during the coronavirus disease 2019 pandemic. During the pandemic, many clinical practices rapidly transitioned to telemedicine, which enabled social distancing and offered new opportunities to bridge geographic barriers to medical care. Prior studies have successfully demonstrated the feasibility and acceptability of electronic health technologies including telemedicine^3^, ^4^, ^5^, ^6^ within the HCV care cascade, but acceptability among hard-to-reach populations is unclear. We investigated acceptability and experiences with telemedicine among a marginalized population undergoing community-based HCV screening in an urban setting.

Between December 2020 and October 2021, we relied on convenience sampling to recruit people experiencing homelessness and people who inject drugs (via posted flyers, street outreach, and informal community referrals) to participate in eligibility screening for a trial study of community-based point-of-diagnosis HCV treatment in San Francisco, CA, USA (NCT03987503). Adults reporting lifetime injection drug use or receipt of a blood transfusion before 1992 and willing and able to provide consent were provided diagnostic HCV screening and a staff administered a close-ended questionnaire (Supplemental Materials). Individuals were excluded if they had been tested for HCV infection in the prior 6 months or if they reported HCV infection with prior incomplete or unsuccessful antiviral treatment. All study materials were approved by the university’s institutional review board. Results reported herein focus on items related to mobile devices and internet access and usage, and experience and satisfaction with prior telemedicine-delivered care. Measurements of telemedicine experience, satisfaction, and concerns are included in Supplemental Materials. Descriptive analyses included median (interquartile range) and frequency (percentage). We compared those with prior telemedicine experience to those without using the Kruskal-Wallis test for continuous variables and the chi-squared or Fisher exact test for categorical variables. We used logistic regression to identify correlates of prior telemedicine use. A second model estimated the association between prior telemedicine use and interest in future telemedicine use. Models were adjusted for age based on its association with telemedicine experience and acceptability.^7^^,^^8^ Models were also adjusted for variables identified to be statistically significant on univariate analysis. All analyses were performed using STATA statistical software package version 14 (STATACorp LP, College Station, TX).

Of the 291 people screened, 290 were eligible and completed the questionnaire (Table). Overall, most (78%) were male, 51% non-Hispanic White, 67% reported injecting drugs within the last 6 months and 89% were unstably housed with 41% living outside or in a vehicle. HCV antibody was reactive in 58%, and among them, 53% had detectable HCV RNA. Access to a smartphone was high (85%) though only 26% had personal wireless internet and most used their cellular network (73%). Ninety-three (32%) had previously participated in a telemedicine visit, 30% telephone only, 25% video only, and 45% both telephone and video; 86% found their telephone visit to be somewhat or extremely helpful and 94% reported the same for their video visit. Additional results regarding telemedicine experience are summarized in Supplemental Materials. Among those with prior telemedicine experience, the most common reasons for visits were primary care (N = 71), mental/emotional health (N = 31), and substance use treatment (N = 23). Compared to those without prior telemedicine use, a greater proportion of those with prior telemedicine use rented or owned their home (8% vs 18%, *P* = .01), had been offered a telemedicine appointment (11% vs 100%, *P* < .001), and expressed being somewhat or very interested in a future telemedicine appointment (64% vs 84%, *P* = .001); however, interest was high in both groups.

**Table:** Characteristics of Study Population

Characteristic	Total population (N = 290)	No prior telemedicine visit (N = 197)	Prior telemedicine visit (N = 93)	*P* value
Age, (median, interquartile range), y	45 (35–55)	44 (35–54)	46 (36–58)	.45
Sex, male [*N* (%)]	227 (78)	156 (79)	71 (76)	.58
Race/ethnicity [*N* (%)], (N = 285)		(N = 195)	(N = 90)	.55
White, non-Hispanic	145 (51)	102 (52)	43 (48)	
Black, non-Hispanic	90 (32)	57 (29)	33 (37)	
Hispanic	21 (7)	14 (7)	7 (8)	
Other	29 (10)	22 (11)	7 (8)	
HCV infection [*N* (%)]				
Reactive HCV antibody	167 (58)	113 (57)	54 (58)	.91
HCV RNA detected^a^	88 (30)	63 (32)	25 (27)	.38
Injection drug use in prior 6 mo [*N* (%)]	195 (67)	131 (67)	64 (69)	.69
Current housing [*N* (%)]				.02
Rent or own	33 (11)	16 (8)	17 (18)	
Temporary housing^b^	138 (48)	92 (47)	46 (49)	
Outdoors or in vehicle	119 (41)	89 (45)	30 (32)	
Smartphone use in the past month [*N* (%)]	246 (85)	162 (82)	84 (90)	.07
Access to personal WiFi [*N* (%)]	76 (26)	46 (23)	30 (32)	.11
Previously offered a telemedicine visit [*N* (%)]	114 (39)	21 (11)	93 (100)	<.001
Telemedicine interest [*N* (%)]				.001
Very interested	83 (29)	52 (26)	31 (33)	
Somewhat interested	121 (42)	74 (38)	47 (51)	
Not interested	56 (19)	42 (21)	14 (15)	
Very not interested	30 (10)	29 (15)	1 (1)	
Telemedicine interest [*N* (%)]				.001
Interested	204 (70)	126 (64)	78 (84)	
Preferred type of visit [*N* (%)] (N = 289)				.50
In person at clinic	202 (70)	136 (69)	66 (72)	
Telemedicine, by phone	48 (17)	36 (18)	12 (13)	
Telemedicine, by video	39 (13)	25 (13)	14 (15)	

a HCV RNA was checked only in those with reactive HCV antibody.

b Temporary housing = single room occupancy, hotel room, staying at shelter, staying with friend, treatment/transitional housing.

When adjusting for age and smartphone access, those living in temporary housing and those living outdoors or in a vehicle had a lower odds of prior telemedicine use (odds ratio 0.4; 95% confidence interval 0.2–0.9 and odds ratio 0.3; 95% confidence interval 0.1–0.7, respectively) than those with stable housing (rent or own; Table A1). We found having had prior telemedicine use had 2.8 greater odds of interest in a future telemedicine use (*P* = .001) compared to no prior telemedicine use when adjusting for age and smartphone access (Table A2).

Despite overall high general interest in telemedicine and high satisfaction among individuals with prior experience, when asked about their *preferred method of care* in the future, most (70%) selected in-person clinic visits, with fewer selecting phone (17%) or video (13%) visits (Table). Participants cited a variety of concerns with telemedicine visits. Several reported a lack of phone/computer and discomfort with technology, while a few reported privacy concerns and other reasons including unreliable or nonsecure internet (data not reported). Many described in-person visits as more personal and more detailed due to the ability to perform vitals and exam.

In summary, within our sample predominantly consisting of people experiencing homelessness and recently injecting drugs, we found that access to digital technology was high, roughly a third had prior experience with telemedicine-delivered care, and interest in future telemedicine care was high. Prior telemedicine use was more likely among those with stable housing. Interest in future telemedicine visits was high overall, although when given the choice most preferred care delivered in-person for future medical care. No significant differences were observed related to HCV status.

As with other chronic diseases, the coronavirus disease 2019 pandemic has had a profound impact on care for HCV patients,^6^ and has disproportionately affected people experiencing homelessness.^9^ For the general population, telemedicine provided an effective solution to overcome geographic barriers to access care, including HCV care.^6^ Our study shows that among a socially marginalized population, while interest and satisfaction may be high, telemedicine may not be a replacement for in-person care. Relying too heavily on telemedicine for marginalized populations may perpetuate healthcare disparities, as has also been demonstrated in other studies.^8^^,^^10^ More research attention is needed to document the impact of telemedicine-delivered care on the health and well-being of people experiencing social marginalization. This study provides an important perspective from individuals who are often overlooked.

Efforts to understand and address barriers to telemedicine use among marginalized populations, particularly those with untreated HCV, must be prioritized to prevent disparate access as telemedicine becomes a primary modality of healthcare delivery. Prior HCV care studies,^4^^,^^5^ as well as others have shown interventions and programs specifically focused on bringing telemedicine to these medically underserved populations at high risk for nonadherence are successful when developed through thoughtful and creative patient-centered approaches.^4^^,^^5^ Following a community-engaged approach when developing programs delivering telemedicine can help identify when and how telemedicine can complement other available care delivery modes.

## Supplementary Materials

Tables A1 and A2
