# Supplementary material for: Telemedicine Acceptability and Experience in a Marginalized Population at Risk for Hepatitis C Virus
Source: Gastro Hep Adv. 2023 Sep 28;3(1):64–6. doi: 10.1016/j.gastha.2023.09.010 (PMC11307729; doi:10.1016/j.gastha.2023.09.010)
Supplement: Tables A1 and A2 [file mmc1.docx]

**Supplemental Table 1: Logistic Regression Models to Identify Factors associated with Prior Telemedicine Experience, N=290***

| **Univariate Analysis** | | | | **Multivariable Analysis** | | | |
| --- | --- | --- | --- | --- | --- | --- | --- |
| **Variable** | **Odds Ratio** | **95% CI** | ***P*-value** | **Variable** | **Odds Ratio** | **95% CI** | ***P*-value** |
| Age | 1.0 | 0.9 to 1.0 | .47 | Age | 1.0 | 0.9 to 1.0 | .45 |
| Gender, female | 1.2 | 0.6 to 2.1 | .58 |  |  |  |  |
| Race and ethnicity, White ref (N=285)  Black or AA  Hispanic  Other** | 1.4  1.2  0.8 | 0.8 to 2.4  0.4 to 3.1  0.3 to 1.9 | .27  .73  .55 |  |  |  |  |
| HCV Ab, reactive | 1.0 | 0.6 to 1.7 | .91 |  |  |  |  |
| HCV RNA, positive | 0.8 | 0.4 to 1.4 | .38 |  |  |  |  |
| Housing, rent/own ref  Temporary housing  Outdoors/vehicle | 0.5  0.3 | 0.2 to 1.0  0.1 to 0.7 | .06  .005 | **Housing, rent/own ref**  **Temporary housing**  **Outdoors/vehicle** | **0.4**  **0.3** | **0.2 to 0.9**  **0.1 to 0.7** | **.04**  **.005** |
| Smart phone access | 2.0 | 0.9 to 4.4 | .08 | Smart phone access | 1.8 | 0.8 to 3.9 | .15 |
| Personal WiFi access | 1.6 | 0.9 to 2.7 | .11 |  |  |  |  |
| Recent IDU | 1.1 | 0.7 to 1.9 | .69 |  |  |  |  |

CI = confidence intervals, ref = reference, AA = African American, HCV = hepatitis C virus, Ab = antibody, IDU = injection drug use

*Unless otherwise indicated

**Other = Asian, Native American/American Indian, Native Hawaiian or Pacific Islander, Mixed, Other, Decline to Answer

**Supplemental Table 2: Logistic Regression Models to Identify Factors associated with Interest in Telemedicine, N=290***

| **Univariate Analysis** | | | | **Multivariable Analysis** | | | |
| --- | --- | --- | --- | --- | --- | --- | --- |
| **Variable** | **Odds Ratio** | **95% CI** | **p-value** | **Variable** | **Odds Ratio** | **95% CI** | **p-value** |
| Age | 1.0 | 0.9 to 1.0 | 0.42 | Age | 1.0 | 0.9 to 1.0 | 0.60 |
| Gender, female | 1.6 | 0.8 to 3.1 | 0.15 |  |  |  |  |
| Race and ethnicity, White ref (N=285)  Black or AA  Hispanic  Other | 0.8  1.1  1.6 | 0.5 to 1.5  0.4 to 2.9  0.6 to 4.2 | 0.55  0.92  0.33 |  |  |  |  |
| HCV Ab, reactive | 1.0 | 0.6 to 1.6 | 0.90 |  |  |  |  |
| HCV RNA, positive | 1.4 | 0.8 to 2.5 | 0.25 |  |  |  |  |
| Housing, rent/own ref  Temporary housing  Outdoors/vehicle | 1.4  1.1 | 0.6 to 3.1  0.5 to 2.4 | 0.46  0.88 |  |  |  |  |
| Smart phone access | 1.8 | 0.9 to 3.5 | 0.08 | Smart phone access | 1.6 | 0.8 to 3.2 | 0.17 |
| Personal WiFi access | 1.0 | 0.5 to 1.7 | 0.89 |  |  |  |  |
| Recent IDU | 1.2 | 0.7 to 2.1 | 0.44 |  |  |  |  |
| Prior telemed visit | 2.9 | 1.6 to 5.5 | 0.001 | **Prior telemed visit** | **2.8** | **1.5 to 5.3** | **0.001** |

CI = confidence intervals, ref = reference, AA = African American, HCV = hepatitis C virus, Ab = antibody, IDU = injection drug use

*Unless otherwise indicated

**Other = Asian, Native American/American Indian, Native Hawaiian or Pacific Islander, Mixed, Other, Decline to Answer
