# Supplementary material for: Telemedicine Acceptability and Experience in a Marginalized Population at Risk for Hepatitis C Virus
Source: Gastro Hep Adv. 2023 Sep 28;3(1):64–6. doi: 10.1016/j.gastha.2023.09.010 (PMC11307729; doi:10.1016/j.gastha.2023.09.010)
Supplement: Supplemental Materials [file mmc2.docx]

**Questionnaire Administration**

Research staff administered a closed-ended questionnaire to participants. Survey questions were read to participants and responses were filled in by research staff. Questionnaires included information about device and internet access and use, prior experience with telemedicine, interest in telemedicine, concerns about telemedicine, and preferred method of care delivery or future medical care.

**Measurements of Telemedicine Experience and Satisfaction**

Participants were asked “Have you ever participated in a telemedicine visit with a healthcare provider over the phone or online instead of in clinic?” Participants who responded with “yes” were then asked “Overall, how was the telemedicine visit experience(s)?” Response options included “extremely helpful”, “somewhat helpful”, “somewhat unhelpful” and “extremely unhelpful”.

**Additional Results of Telemedicine Experience Questions and Overall Satisfaction**

Participants’ experience with telemedicine was summarized as “high satisfaction” based on the responses to the statement “please rate the level to which you agree or disagree, (choices included “strongly agree”, “agree” “neutral”, “disagree”, and “strongly disagree”) with the following statements about your telemedicine video visit(s)”:

- I thought the telemedicine visit was easy to use – 82% agree or strongly agree
- I think I would need the support of a technical person to be able to use video visits in the future – 75% disagree or strongly disagree
- I found scheduling the video visit easy enough – 87% agree or strongly agree
- I was able to discuss my medical concerns/problems well enough during the video visit – 83% agree or strongly agree
- I feel that telemedicine video visits are a convenient form of medical care for me – 83% agree or strongly agree
